# The Physician’s Electrical Outfit and Its Daily Use1Read at the annual meeting of the Medical Society of the County of Allegany, June 13, 1903.

**Published:** 1903-09

**Authors:** Joseph C Clark

**Affiliations:** Olean, N. Y.


### The Physician's Electrical Outfit and its Daily Use.[1]

BY JOSEPH C CLARK, M. D., Olean, N. Y.

IN READING this paper upon the electrical outfit and its daily use, I wish to give warning that I have no new discoveries to present, and no startling cures with which to arouse your doubts. My idea is to interest the sceptical, to encourage the doubting, and to add moral support to all those who have been using elec-

1. Read at the annual meeting of the Medical Society of the County of Allegany, June 13, 1903.

tricity as an aid to their skill in treating medical and surgical conditions. Twenty-five centuries ago the Phœnecian voyagers, who sailed upon the desolate shores of the Baltic Sea, brought back a substance greatly prized by the ancients for its fair color and transparency. It was amber or electron. But to Thales, the Benjamin Franklin of Miletus, it possessed a mysterious value. He discovered that amber, when rubbed, had the power of attracting to itself various light articles, as if endowed with life. His discovery was the first step in the great science of electricity. The centuries passed, and not until the XVI. did any new developments of importance take place. The improvements and advances made in the uses of electricity recently, are such as to entitle the XX. century to be called the era of electricity, and we do not think the physician alone, of educated men, should remain isolated from the general advance, or lag behind in this great branch of scientific thought and practice.

A noted Englishman, Edward Owen, recently made the statement that "the medical use of electricity had fallen largely into the hands of quacks". "This might have been said truthfully 25 years ago", replies Dr. Rockwood, of New York, "but not now, because there are hundreds of reputable and able men, both here and abroad, who fully recognise the influence of the various methods of electrisation over general and local nutrition. As to those who have no knowledge of electricity, and so have no faith, so much the worse for them". The trouble is the *x*-ray and static electricity have been lauded too much as cure-alls to satisfy rational medical conservatism.

The proper selection of apparatus and electrodes is important to the physician, in order to afford his patient all the benefits of this variable and effective form of electricity,—galvanic batteries from two dozen fruit jars, with attachments, are as effective, as storage or dry-cell batteries. But the dry-cell battery's reputation is fully established, having been adopted by the United States Government; it insures compactness and durability, is always ready for work and is free from liquid. The advantage of a current controller is very evident; all the cells work together and at once; under the old method some cells naturally gave out sooner than others; again, under its influence there is no shock to the patient in increasing the current, which is always completely and simply under control, and this advantage in certain nervous diseases is preëminent. A milliampèremeter is always to be desired, to intelligently measure the galvanic current, which seldom, if ever, should be given above 10 milliampères, nor for longer than 10 minutes. The dry-cell faradic battery is compact, always works, is easily changed when cells show fatigue, and as far as my investigations go, is all that any practitioner needs. This current is safe and never is followed by reaction, like the galvanic battery which will often give a patient a decided nervous time if overdone in its application.

Regarding the static apparatus, my preference is for 10 revolving plates and a motive power, preferably a water motor, with which the operator has the apparatus under thorough control, but which is not the case always with an electric motor. The electric motor must run at full speed always, and the *x*-ray developed must of necessity make a soft tube hard very soon. This can be controlled somewhat by having a tube with a movable target, but even then the life of a tube is much shortened.

Some outfits are made to develop large currents by being turned by hand,—namely, the Wagner mica plate, a very elegant apparatus, and I am not certain but in a few years mica plates will supplant the glass wheels. All depends upon their durability. A static instrument that sparks across in the case is imperfect and should not be accepted until the defect is remedied. One should know his static machine as well as he knows his horse, and not be afraid of it; he should master its mechanical details and so be ever ready for the off days that every good static indulges in, in every day practice. The poles of the static current have a common effect. The usual pole connected with the platform is the negative.

Let us dissect out the sciatic nerve at its exit from the pelvis and leave it connected with the leg below. Now apply a positive electrode to the nerve where cut, and an indifferent electrode to the nerve as it turns around the head of the fibula, then connect it to a galvanic, faradic or static current, and an energetic contraction occurs; a much more powerful current is necessary if the poles are applied without regard to the nerve supply of a muscle. If the positive pole of the galvanic current is attached to the nerve above, and the negative below, when the closure of the circuit occurs, the strongest contraction takes place at the negative or cathode pole; so, upon opening the current the strongest contraction occurs at the anode or positive pole; the reverse of this occurring constitutes the reaction of degeneration of Erb.

The physiological or functional properties of galvanic, faradic and static electricity are very much the same, yet in my experience results are best when I keep the idea of galvanic for nerve trouble and adhesions, faradic for muscle and nutrition, and static for both. It has been claimed that the negative pole retards nerve degeneration and the positive accelerates it. The negative pole dissolves exudates and exerts what may be termed reabsorptive action. I have a decided preference for the negative pole, and the dissolving action of the negative pole is most marked in the use of bare electrodes like needles and sounds. The action of the positive pole is caustic or astringent, depending upon the strength of the current. The negative pole produces softening and relaxation, and stimulates absorption of effete products.

There are many diseased conditions of the human body very much benefited by the use of electricity. I will consider a few from a personal experience standpoint, and some from reported cases. First, we have paralysis, local or central, or partial. In either condition the muscles show a marked progress toward loss of tone, and flabbiness. This can be prevented and tone built up by the use of either the galvanic, faradic, or static currents. The degeneration of local nerves can be proven by our galvanic test. Pain due to nerve, muscle, or any cause can often be very much relieved, if not stopped entirely. Here we may include nervous neuralgia, rheumatic and inflammatory conditions, recent sprains, and lumbago. Fibrous anchylosis and tendons attached to sheaths are very much helped by any one of the three currents and more particularly by the galvanic with a salt solution upon the anode or positive pole.

The many uses of static electricity in combination with the *x*-ray are increasing monthly, and we find the static current in neurasthenia is as good as any agent known, in the treatment of many of these unfortunate conditions. The tonic effect of static electricity does not exceed that of the galvanic or faradic; the careful and thorough use of the galvanic and faradic current constitutes one of the greatest tonics and stimulants that can be brought to bear upon the human system. It awakens torpid conductivity of every nerve thread in the body and its greatest benefits can be demonstrated in the aged in whom loss of superficial sensation can be proved, only to quickly return after a few treatments by the galvanic and faradic currents. The galvanic current should be used never longer than ten minutes to begin with, for fear of reaction, such as chills and nervousness. But it is some trouble to disrobe, and the exposure often deters the medical man from using these currents. The static apparatus here comes in as a quick and convenient method of giving electricity, and, as an enthusiastic patient once remarked, "it is good for whatever ails you".

While the constant current has proved so very useful to the medical profession for diagnosis, for stimulating nerve and muscle, for cauterisation, and for electrical endoscopy, we must not neglect its cataphoric effect by which remedial agents are diffused through the tissues and fluids of the body, to improve nutrition, to produce anesthesia, to relieve pain, to destroy germs, to modify morbid processes, and to make soluble chemical combinations with deleterious substances which collect in the organism. The medicines used are the iodides of potassium, sodium or lithium, normal salt, iodol or diluted tincture of iodine, upon positive or anode; chloride, benzoate or citrate of lithium, all very soluble, in gout or rheumatism. These remedies are lauded very much in the treatment of Graves's disease or exophthalmic goitre and there must be something in this claim, for I have used, and always do use, the galvanic in goitre with good, and often marked results. Here the positive pole should be placed over the gland or any enlarged gland for five minutes.

The uses of the *x*-ray in diseased conditions has been very much overworked in my judgment. There is, however, one condition that yields to its influence, and that is the chronic superficial forms of lupus. The deeper forms will often apparently heal only to reappear. The indications for its use are: to relieve pain, to lessen edema, to promote absorption, to act as a parasiticide, to act as a bactericide, to cause the destruction of malignant growths, to limit the extension of malignant growths, to prevent the recurrence of malignant growths; these are but some of the indications for the employment of this agent. From a long list of reports I have collected the following *cures,*—lupus, superficial; lupus, deep; tuberculosis of the glands, lungs, skin or bones; epithelioma; goiter; rodent ulcers; eczema; carcinoma; osteosarcoma; neuralgia of the ovaries; removing moles, and other similar cures. In conclusion, I hope I have interested the sceptical, encouraged the doubting and given moral support to all those who use electricity as one of the many methods in their practice of the healing art.

